# My Address at Milwaukee

**Published:** 1881-02

**Authors:** E. W. Berridge

**Affiliations:** London


### MY ADDRESS AT MILWAUKEE.

BY E. W. BERRIDGE, M.D., LONDON.

My address at Milwaukee has had at least *one* good result; it has compelled the mongrels to exhibit themselves in their true colors and display their irreconcilable hatred for the homœopathy of Hahnemann. Their denunciations and misrepresentations of myself, I treat with the silent contempt they deserve; I shall only deny the falsehood they have circulated about two of my friends. It has been publicly asserted that I prepared and read my address, at Milwaukee, at the instigation of Drs. Ad. Lippe and T. P. Wilson. This assertion, I pronounce *a deliberate and malicious fabrication.*

The only individual on either side of the "pond" who knew of the contents of my address was Dr. H. N. Guernsey, of Philadelphia. When Dr. T. P. Wilson heard of my intended visit, he wrote me a letter of welcome, telling me that I must be prepared to address the Institute; *but made no other suggestions.* Shortly afterward, Dr. H. N. Guernsey visited me, I read him the letter of Dr. Wilson, and asked what kind of address would be expected; whether a theoretical essay, some clinical cases, or what? He suggested a paper inculcating the study of Hahneman's "Organon." I coincided with his views and wrote my paper. When I saw Dr. Guernsey, again, at my house, I read it to him. He made no further suggestions, but thoroughly approved of it, saying that he would vote for the printing of one thousand copies for distribution; but his absence from the meeting prevented him from carrying out his intention. The address was, with a few merely verbal alterations, precisely the same as read to Dr. Guernsey; for its sentiments *I alone* am responsible.

Dr. T. P. Wilson knew so little of it beforehand, that, when asked about it as president, he was unable to give even the title. While staying in Philadelphia, as Dr. Ad. Lippe's guest,

I intended to read the address to him and obtain his opinion and advice; but day after day passed by without a convenient opportunity arising; so (fortunately, as it happened), I did not read it to him.

From the above refutation of *one* of the falsehoods circulated by the mongrels, Homœopathicians will be able to form an estimate of their candor in other matters. *Ex uno disce omnes.*

